# Analysis of SNP Array Abnormalities in Patients with *DE NOVO* Acute Myeloid Leukemia with Normal Karyotype

**DOI:** 10.1038/s41598-020-61589-9

**Published:** 2020-04-03

**Authors:** Mariam Ibáñez, Esperanza Such, Esther Onecha, Inés Gómez-Seguí, Alessandro Liquori, Jorge Sellés, David Hervás-Marín, Eva Barragán, Rosa Ayala, Marta LLop, María López-Pavía, Inmaculada Rapado, Alex Neef, Alejandra Sanjuan-Pla, Claudia Sargas, Elisa Gonzalez-Romero, Mireia Boluda-Navarro, Rafa Andreu, Leonor Senent, Pau Montesinos, Joaquín Martínez-López, Miguel Angel Sanz, Guillermo Sanz, José Cervera

**Affiliations:** 1 Hematology Service, Hospital Universitario y Politécnico La Fe, Valencia, Spain; 2 Centro de Investigación Biomédica en Red de Cáncer, Madrid, Spain; 3 Departamento de Ciencias Biomédicas. Facultad de Ciencias de la Salud. Universidad CEU Cardenal Herrera, Valencia, Spain; 4 Hospital 12 de Octubre, Madrid, Spain; 5 Grupo de Investigación en Hematología, IIS La Fe, Valencia, Spain; 6 Array’s Unit. Instituto Investigación Sanitaria Fundación La Fe, Valencia, Spain; 7 Biostadistic Unit. Instituto Investigación Sanitaria Fundación La Fe, Valencia, Spain; 8 Laboratory of Molecular Biology, Department of Clinical Chemistry, University Hospital La Fe, Valencia, Spain; 9 Hematology Service, Hospital General, Valencia, Spain; 10 Department of Medicine. University of Valencia, Valencia, Spain; 11 Genetics Unit, Hospital Universitario y Politécnico La Fe, Valencia, Spain

**Keywords:** Haematological cancer, Haematological cancer, Haematological cancer, Haematological cancer, Acute myeloid leukaemia

## Abstract

Nearly 50% of patients with *de novo* acute myeloid leukemia (AML) harbor an apparently normal karyotype (NK) by conventional cytogenetic techniques showing a very heterogeneous prognosis. This could be related to the presence of cryptic cytogenetic abnormalities (CCA) not detectable by conventional methods. The study of copy number alterations (CNA) and loss of heterozygozity (LOH) in hematological malignancies is possible using a high resolution SNP-array. Recently, in clinical practice the karyotype study has been complemented with the identification of point mutations in an increasing number of genes. We analyzed 252 *de novo* NK-AML patients from Hospital La Fe (n = 44) and from previously reported cohorts (n = 208) to identify CCA by SNP-array, and to integrate the analysis of CCA with molecular alterations detected by Next-Generation-sequencing. CCA were detected in 58% of patients. In addition, 49% of them harbored CNA or LOH and point mutations, simultaneously. Patients were grouped in 3 sets by their abnormalities: patients carrying several CCA simultaneously, patients with mutations in *FLT3, NPM1* and/or *DNMT3A* and patients with an amalgam of mutations. We found a negative correlation between the number of CCA and the outcome of the patients. This study outlines that CCA are present in up to 50% of NK-AML patients and have a negative impact on the outcome. CCA may contribute to the heterogeneous prognosis.

## Introduction

Acute myeloid leukemia (AML) is a heterogeneous disease that represents the most frequent type of acute leukemia in adults. Conventional cytogenetic studies have shown that cytogenetic alterations are frequent in AML, being useful for diagnosis, classification and prognosis purposes^1^. However, almost 50% of the patients present an apparently normal karyotype (NK) by conventional cytogenetic techniques. In addition, the prognosis of these patients is very heterogeneous suggesting that cryptic alterations not detectable with such conventional methods may be able to develop the disease, having different prognostic implications.

The high resolution single-nucleotide polymorphism array (SNP-A) is a powerful tool for the study of copy number alterations (CNA), loss of heterozygosity (LOH) and chromothripsis in hematological malignancies^2,3^. Moreover, the study of matched germline/tumor tissues allows detecting acquired alterations and, therefore, those involved in the leukemogenesis. During the last decade, several studies have been carried out using SNP-A with the aim of improving the cytogenetic characterization of AML patients. Most of these studies have only analyzed a few dozens of patients, and those that have involved a greater number of patients, either did not analyze systematically matched sample (tumor/germline) or used low density arrays^2–13^. In general, these studies have shown that acquired non-recurrent submicroscopic variations are frequent in patients with *de novo* AML, many of them involving disease-relevant genes, such as *TP53* or *FLT3*. In addition, some of these studies have observed a negative effect on the outcome of these patients, but not in a homogenous manner.

Likewise, in clinical practice, the study of the karyotype by G-bands has recently been complemented with the identification of point mutations in an increasing number of genes. Very recently, the classification of patients with AML has been redefined in 3 different risk groups based on their molecular alterations^14^. However, in spite of all these efforts, the biological knowledge that underlies the leukemogenic process of these patients remains unclear, especially in those who do not carry cytogenetic alterations.

In this study SNP-A, NGS and survival data were analyzed from 120 paired samples (somatic/germinal) of *de novo* NK-AML patients to identify acquired cryptic cytogenetic abnormalities by SNP-A and to integrate analysis of cryptic cytogenetic alterations with molecular alterations detected by targeted sequencing. Finally, to extend our knowledge, we compared our SNP-A findings with data from previously published series, up to a total of 252 patients with *de novo* NK-AML.

## Methods

### Patients and samples

Patients diagnosed with *de novo* AML in the Hospital Universitari i Politècnic La Fe (n = 32) and in Hospital Universitario 12 de Octubre (n = 12) with available tumor and germline DNA sample were selected for this study (La Fe Cohort). DNA was provided by Biobank La Fe. Tumor DNA was obtained from bone marrow cells at diagnosis. Matched germline DNA was obtained from peripheral blood at morphological and molecular complete remission time or saliva. Conventional cytogenetics (with a banding for normal karyotypes between 450 and 550 BPHS), FISH and NGS were performed in every case, as well as tests for mutation detection of *FLT3-ITD* and D835, as previously described^14^. Patients were enrolled in consecutive multicenter PETHEMA trials (PETHEMA 2007-*NCT02006004* and PETHEMA-LMA10-*NCT01296178*). Clinical data, as well as treatment outcome and follow-up, were collected prospectively. The last update on clinical data was performed on December 2017. This study was approved by the Research Ethics Board of IISLAFE (No. 2012/0175) and informed consent in accordance with the Declaration of Helsinki was obtained before taking sample for genetic and genomic research.

### SNP-A

Samples (500 ng) were genotyped with Cytoscan HD (Affymetrix) according to manufacturer’s protocol (Affymetrix Santa Clara, C.A., U.S.A.). DNA copy number and paired LOH analysis were performed using the Genotyping Console and the Chromosome Analysis Suite (ChAS) software (Affymetrix). Filters applied for the detection of segmental CNA were ≥20 consecutive markers in a region of at least 50 Kb, and for regions of CN-LOH, ≥100 markers in at least 5000 Kb. We considered as cryptic aberrations all the aberrations not detected by daily cytogenetic test (G banding pattern and by FISH), independently of their size. All abnormalities found in the remission sample were ruled out and assumed as non-somatic. In addition, to exclude germ line alterations every potential abnormality was checked in the Database of Genomic Variants (http://projects.tcag.ca/variation) to determine significant overlapping with polymorphic variations reported. Size, position, and location of genes were identified with UCSC Genome Browser (http://genome.ucsc.edu/). The human reference sequence used for alignment was the GRCh37/hg19assembly.

### SNP-A data from other AML series

We compared our results with other AML series, namely AML cases from The Cancer Genome Atlas (TCGA) Network with publicly available SNP-A and NGS data (n = 76), the series of Krönke J, *et al*. (n = 53), the series of Akagi *et al*. (n = 30) and the series of Koren-Michowitz M, *et al*. (n = 49)^4–6,15^. Lesions found in the series of Krönke J, *et al*., Akagi T *et al*. and Koren-Michowitz M, *et al*. were listed in the corresponding report^4–6^. In total, we analyzed data from 252 patients *de novo* NK-AML (Supplementary Table 1).

Conversion of hg18 to version hg19 was done in these cases using the “Batch Coordinate Conversion (liftOver)” Tool from the UCSC Genome Browser, with a minimum ratio of bases that remapped >0.95. Germline alterations were excluded from the analysis by visual inspection and by comparison with the polymorphic variations reported in the Database of Genomic Variants (http://projects.tcag.ca/variation/).

### NGS targeted sequencing

The complete coding regions of the following genes were sequenced, *BCOR, BRAF, CDKN2A, CEBPA, DNMT3A, ETV6, EZH2, GNAS, LUC7L2, NF1, PHF6, PTPN11, RAD21, RPS14, SF1, SF3A1, SMC3, SPARC, SRSF2, STAG2* and *ZRSR2*, as well as, the hotspot regions of *ASXL1, MPL, NPM1, JAK2, KRAS, NRAS, TET2, U2AF1, KIT, IDH1, RUNX1, IDH2, SETBP1, TP53, WT1, CBL*, and *SF3B1*, using an amplicon panel (Ampliseq, Life Technologies) with an Ion Torrent Proton according to the manufacturer’s instructions. Primary bioinformatic analysis was performed using an in-house protocol and variants were selected based on VAF ≥ 1%, its absence in the healthy population (UCSC Common SNPs; MAF < 0.01) and its putative effect on the protein.

### Statistical analysis

Data were summarized using mean (standard deviation) and median (1st and 3rd quartile) in the case of continuous variables and with relative and absolute frequencies in the case of categorical variables. Association of the different clusters of samples with survival was assessed using Cox regression models. All P values reported are two-sided. Multivariable time-to-event analyses were performed using elastic net penalized cox regression models and random forest survival models. For the elastic net analysis, an initial alpha value of 0.2 was selected and 100 replicates of 5-fold cross validation were used to estimate the penalization parameter in the elastic net models following the one-standard-error rule. Alternative alpha values were used to assess the stability and robustness of the estimates. Random forest models were adjusted with 1000 trees and p/3 variables randomly selected as candidates for each node split. All statistical analyses were performed using R (version 3.3.2) and the R-packages randomForestSRC (version 2.4.2) and glmnet (version 2.0–5).

### Ethics approval and consent to participate

This study was approved by the Research Ethics Board of IISLAFE (No.2012/0175) and informed consent in accordance with the Declaration of Helsinki was obtained before taking sample for genetic and genomic research.

## Results

### Identification of CNA and CN-LOH by SNP-A analysis among all cohorts together (n = 252)

**Figure 1:**
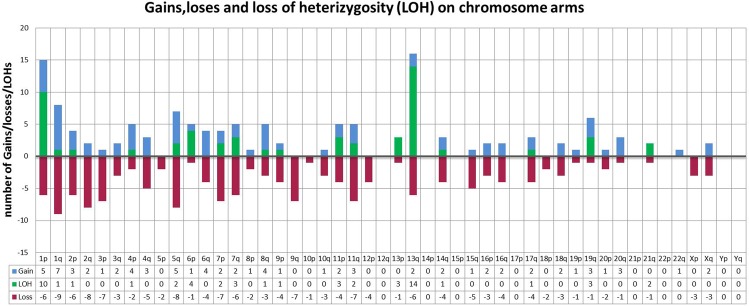
Distribution of Gains, losses and CN-LOH among all cohorts together (n = 252). Gains appear in blue; losses in red and CN-LOH in green.

**Figure 2:**
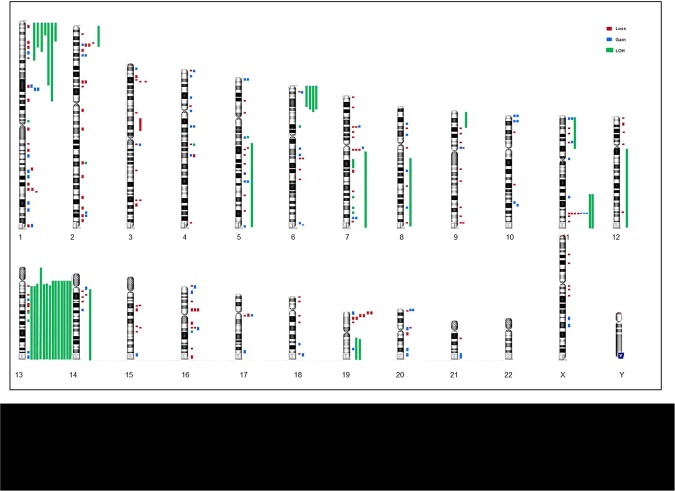
Karyogram of AML according to SNP-A analyses among all cohorts (n = 252). Coloured bars depict the extension of abnormalities. Gains appear in blue at the right side of each chromosome; losses in red and CN-LOH in green.

A total of 120 patients were included in this study [LaFe cohort (n = 44) and TCGA cohort (n = 76)] after a statistical analysis that established no differences between series of patients. Main clinical and genetic features of patients from both cohorts are summarized in Table 1. In addition, we extended this cohort with data from 132 patients previously reported^3–5^. Among all cohorts together (n = 252), SNP-A analysis revealed 282 cryptic abnormalities in 58% of patients (N = 146), with an average of 2.32 abnormalities/patient (range 1–39). These consisted of 152 heterozygous deletions (54%), 76 duplications (27%) and 54 CN-LOH (19%; 35% of them interstitial). A detailed list of the CNA and CN-LOH found in these series is shown in Table 2 and Fig. 1. Median size of CNA was 2.9 Mb (range 0.11–25.94), 5.3 Mb (range 0.01–28.38) and 18.9 Mb (range 7.9–109.28) for gains, loss and CN-LOH respectively. No statistical differences in size were observed. Losses were distributed virtually across all chromosomes, unlike CN-LOH or gains (Fig. 2). On average, 41.3 genes (range: 1–446) were involved in deleted regions, 517.5 genes (range: 41–1123) in CN-LOH and 73.2 genes (range: 1–452) in gains, with a gene density of 7.8 genes/Mb, 11.5 genes/Mb and 35.28 genes/Mb, respectively. Chromosomes with a greater number of alterations were chromosomes (chr) 1 (n = 38), 2 (n = 20), 5 (n = 17), 7 (n = 22), 11 (n = 21) and 13 (n = 22). In general, the chromosomal alterations involved genes such as *KMT2A, FLT3, ETV6, RUNX1* and *HNPRK*. CN-LOHs were generally concentrated in chr 1, 2, 5q, 7, 11q and 13q, mostly in regions where genes with an impact on the survival of AML patients are located, such as *FLT3* in 13q.

**Table 1:** Main characteristics of our series (n = 120).

Characteristics	Ibáñez, *et al*. (N = 44)	TCGA (N = 76)
Gender	21 Males	39 Males
Median age (range)	46 (20–76)	57.5 (21–88)
Hemoglobin g/dL, median (range)	8.7 (6.1–13.4)	NA
Platelet count x109/L, median (range)	68.5 (8–184)	NA
Leukocyte count x109/L, median (range)	13.7 (0,8–190)	27.35 (0,6–298)
BM Blasts (%), median (range)	66 (20–97)	72 (30–100)
*FLT3*-ITD positive	13 (29%)	20 (27%)
NPM1 mutated	22 (50%)	46 (56%)

**Table 2:** Detailed list of abnormalities found in our series (n = 120).

id	Cohort	Type	Chromosome	CytobandStart	Cytobandend
#12	M Ibañez *et al*.	Loss	3	p25.1	p25.1
#12	M Ibañez *et al*.	Loss	3	p14.2	p12.3
#14	M Ibañez *et al*.	Gain	6	q27	q27
#14	M Ibañez *et al*.	Loss	11	q23.3	q23.3
#17	M Ibañez *et al*.	Gain	11	q23.3	q23.3
#24	M Ibañez *et al*.	Loss	3	p25.1	p25.1
#24	M Ibañez *et al*.	LOH	19	q13.11	q13.43
#26	M Ibañez *et al*.	LOH	1	p36.33	p35.1
#27	M Ibañez *et al*.	Loss	X	q13.1	q13.1
#28	M Ibañez *et al*.	Loss	11	p14.1	p12
#31	M Ibañez *et al*.	Loss	11	q23.1	q23.2
#31	M Ibañez *et al*.	Loss	13	q13.3	q14.11
#31	M Ibañez *et al*.	Loss	16	q12.1	q24.3
#31	M Ibañez *et al*.	Loss	16	p11.2	p11.1
#31	M Ibañez *et al*.	Loss	4	q26	q26
#32	M Ibañez *et al*.	LOH	13	q12.11	q34
#34	M Ibañez *et al*.	Loss	5	q14.3	q33.3
#35	M Ibañez *et al*.	Gain	11	q23.3	q23.3
#36	M Ibañez *et al*.	LOH	13	q12.11	q34
#39	M Ibañez *et al*.	Loss	17	q21.31	q21.31
#42	M Ibañez *et al*.	LOH	1	p36.33	p34.1
#42	M Ibañez *et al*.	LOH	17	q11.2	q25.3
#44	M Ibañez *et al*.	Loss	1	q42.2	q42.2
#44	M Ibañez *et al*.	Loss	17	q25.3	q25.3
#46	M Ibañez *et al*.	Loss	1	q32.1	q32.1
#46	M Ibañez *et al*.	Loss	5	q35.3	q35.3
#46	M Ibañez *et al*.	Loss	7	q21.2	q21.2
#46	M Ibañez *et al*.	Loss	13	q14.11	q14.11
#46	M Ibañez *et al*.	Loss	20	q11.22	q11.22
#46	M Ibañez *et al*.	Loss	2	p14	p14
#46	M Ibañez *et al*.	Loss	2	q22.3	q22.3
#46	M Ibañez *et al*.	Loss	2	q31.3	q31.3
#46	M Ibañez *et al*.	Loss	3	p25.1	p25.1
#46	M Ibañez *et al*.	Loss	9	q21.2	q21.2
#47	M Ibañez *et al*.	Gain	8	q24.21	q24.21
#47	M Ibañez *et al*.	Gain	19	q13.33	q13.43
#47	M Ibañez *et al*.	Gain	22	q13.31	q13.33
#47	M Ibañez *et al*.	LOH	19	q13.11	q13.43
#47	M Ibañez *et al*.	Loss	2	q37.1	q37.3
#49	M Ibañez *et al*.	Gain	11	q23.3	q23.3
#49	M Ibañez *et al*.	LOH	11	q12.3	q25
#50	M Ibañez *et al*.	Loss	7	q34	q34
#50	M Ibañez *et al*.	LOH	7	q31.32	q34
#50	M Ibañez *et al*.	LOH	7	q34	q36.3
#54	M Ibañez *et al*.	LOH	1	q21.1	q44
#55	M Ibañez *et al*.	Gain	18	q21.32	q21.32
#56	M Ibañez *et al*.	LOH	1	p36.32	p13.3
#57	M Ibañez *et al*.	LOH	5	q13.2	q15
#57	M Ibañez *et al*.	LOH	7	p22.1	p21.3
#57	M Ibañez *et al*.	LOH	7	q33	q35.3
#57	M Ibañez *et al*.	LOH	11	q22.1	q22.1
#8	M Ibañez *et al*.	LOH	1	p36.33	p36.13
#8	M Ibañez *et al*.	Loss	1	p34.3	p34.3
#8	M Ibañez *et al*.	Loss	13	q14.11	q14.11
#8	M Ibañez et al.	LOH	13	q12.11	q34
2802	TCGA	Loss	1	p35.2	p35.2
2802	TCGA	Loss	1	q32.1	q32.1
2802	TCGA	Gain	1	q31.1	q31.1
2802	TCGA	Loss	2	p14	p14
2802	TCGA	Loss	2	q11.2	q11.2
2802	TCGA	Loss	2	q14.2	q14.2
2802	TCGA	Loss	2	q37.1	q37.1
2802	TCGA	Loss	5	p12	p12
2802	TCGA	Gain	5	q31.1	q31.1
2802	TCGA	Loss	5	q31.3	q31.3
2802	TCGA	Loss	5	q33.3	q33.3
2802	TCGA	Loss	5	q35.1	q35.1
2802	TCGA	Loss	6	q14.3	q14.3
2802	TCGA	Loss	6	q21	q21
2802	TCGA	Loss	7	p22.3	p22.3
2802	TCGA	Loss	7	p13	p13
2802	TCGA	Loss	7	q31.2	q31.2
2802	TCGA	Gain	8	p21.3	p21.3
2802	TCGA	Loss	8	q22.1	q22.1
2802	TCGA	Loss	8	q22.1	q22.1
2802	TCGA	Loss	10	q21.2	q21.2
2802	TCGA	Loss	10	q22.3	q22.3
2802	TCGA	Loss	10	q24.13	q24.13
2802	TCGA	Loss	11	p15.1	p15.1
2802	TCGA	Gain	11	p11.12	p11.12
2802	TCGA	Loss	14	q12	q12
2802	TCGA	Loss	16	q12.2q13	q12.2q13
2802	TCGA	Loss	18	p11.22	p11.22
2802	TCGA	Loss	18	q23	q23
2802	TCGA	Gain	20	p11.21	p11.21
2802	TCGA	Gain	20	q13.32	q13.32
2802	TCGA	Loss	21	q22.11	q22.11
2802	TCGA	Loss	X	p21.3	p21.3
2802	TCGA	Loss	X	q13.1	q13.1
2802	TCGA	Loss	3	q11.2	q11.2
2802	TCGA	Loss	3	q22.1	q22.1
2802	TCGA	Loss	4	q35.1	q35.1
2802	TCGA	Loss	9	p13.2	p13.2
2802	TCGA	Loss	9	q33.3	q33.3
2811	TCGA	Gain	7	p13	p13
2811	TCGA	Gain	14	q11.2	q11.2
2811	TCGA	Loss	16	p12.13	p12.13
2811	TCGA	Gain	19	p13.3	p13.3
2811	TCGA	Gain	19	q13.43	q13.43
2812	TCGA	Gain	2	p24.1	p24.1
2812	TCGA	Loss	5	q14.3	q14.3
2812	TCGA	Loss	6	p24.3	p24.3
2812	TCGA	Gain	6	q13	q13
2812	TCGA	Loss	13	q11	q11
2812	TCGA	Gain	X	q22.3	q22.3
2824	TCGA	Loss	1	p36.32	p36.32
2825	TCGA	Loss	7	p14.1	p14.1
2826	TCGA	Gain	1	p31.1	p31.1
2831	TCGA	Loss	14	q11.2	q11.2
2833	TCGA	Gain	18	q23	q23
2866	TCGA	Gain	20	q13.33	q13.33
2871	TCGA	LOH	2		
2871	TCGA	Gain	1	q44	q44
2871	TCGA	Gain	3	q26.1	q26.1
2871	TCGA	Gain	4	p15.1	p15.1
2871	TCGA	Gain	4	q13.2	q13.2
2879	TCGA	Gain	4	p15.1	p15.1
2884	TCGA	Loss	1	q25.2	q25.2
2896	TCGA	Loss	16	p13.3	p13.3
2896	TCGA	Loss	19	q13.43	q13.43
2907	TCGA	Loss	1	p12	p12
2907	TCGA	Loss	1	q21.3	q21.3
2907	TCGA	Gain	1	q25.2	q25.2
2907	TCGA	Gain	2	q36.1	q36.1
2907	TCGA	Gain	5	q13.3	q13.3
2907	TCGA	Gain	5	q35.3	q35.3
2907	TCGA	Loss	7	q11.21	q11.21
2907	TCGA	Gain	7	q35	q35
2907	TCGA	Gain	7	p36.12	p36.12
2907	TCGA	Loss	11	q23.3	q23.3
2907	TCGA	Loss	12	p13.33	p13.33
2907	TCGA	Loss	4	q22.1	q22.1
2907	TCGA	Gain	15	q26.3	q26.3
2907	TCGA	Loss	16	q12.2	q12.2
2907	TCGA	Gain	16	q12.2	q12.2
2907	TCGA	Loss	16	q24.1	q24.1
2907	TCGA	Gain	17	q25.3	q25.3
2907	TCGA	Loss	20	p11.21	p11.21
2907	TCGA	Gain	20	q13.2	q13.2
2907	TCGA	Loss	X	q12	q12
2907	TCGA	Loss	9	q34.2	q34.2
2907	TCGA	Loss	15	q14	q14
2907	TCGA	Loss	15	q23	q23
2919	TCGA	Gain	4	q24	q24
2919	TCGA	Gain	7	q11.1	q11.1
2919	TCGA	Gain	8	q11.1	q11.1
2919	TCGA	Gain	8	q21.12	q21.12
2919	TCGA	Gain	X	q23	q23
2921	TCGA	Loss	12	p11.12	p11.12
2921	TCGA	Loss	X	p21.1	p21.1
2922	TCGA	Loss	7	p14.1	p14.1
2924	TCGA	LOH	1	1p	
2934	TCGA	Loss	18	p11	p11
2964	TCGA	Gain	4	q13.2	q13.2
2964	TCGA	LOH	21	21q	
2966	TCGA	Loss	2	p23.3	p23.3
2966	TCGA	Gain	1	p31.1	p31.1
2966	TCGA	Gain	2	p22.3	p22.3
2967	TCGA	Loss	2	q14.3	q14.3
2968	TCGA	Gain	1	p35.1	p35.1
2968	TCGA	Loss	11	q23.3	q23.3
2970	TCGA	LOH	21	21q	
2971	TCGA	Gain	1	p31.1	p31.1
2972	TCGA	Gain	2	p22.3	p22.3
2973	TCGA	Loss	8		
2973	TCGA	Loss	11	q23.3	q23.3
2973	TCGA	Loss	X	p21.1	p21.1
2974	TCGA	Loss	3	q26.1	q26.1
2976	TCGA	Loss	8	p12	p12
2977	TCGA	Loss	1	p22.2	p22.2
2977	TCGA	Gain	16	p13.2	p13.2
2983	TCGA	LOH	11	11p	
2983	TCGA	Loss	4	p16.3	p16.3
2986	TCGA	Gain	1	p31.1	p31.1
2987	TCGA	Loss	7	q11.21	q11.21
2987	TCGA	Loss	17	q11.2	q11.2
2989	TCGA	Loss	14	q12	q12
2992	TCGA	Loss	5	5q	5q
3006	TCGA	Gain	9	p11.2	p11.2
3008	TCGA	Loss	1	q31.3	q31.3
3008	TCGA	Loss	9	p12-p11.2	p12-p11.2
3009	TCGA	Gain	13		
3009	TCGA	Gain	16	p13.3	p13.3

### Correlation with mutations detected by NGS

**Figure 3:**
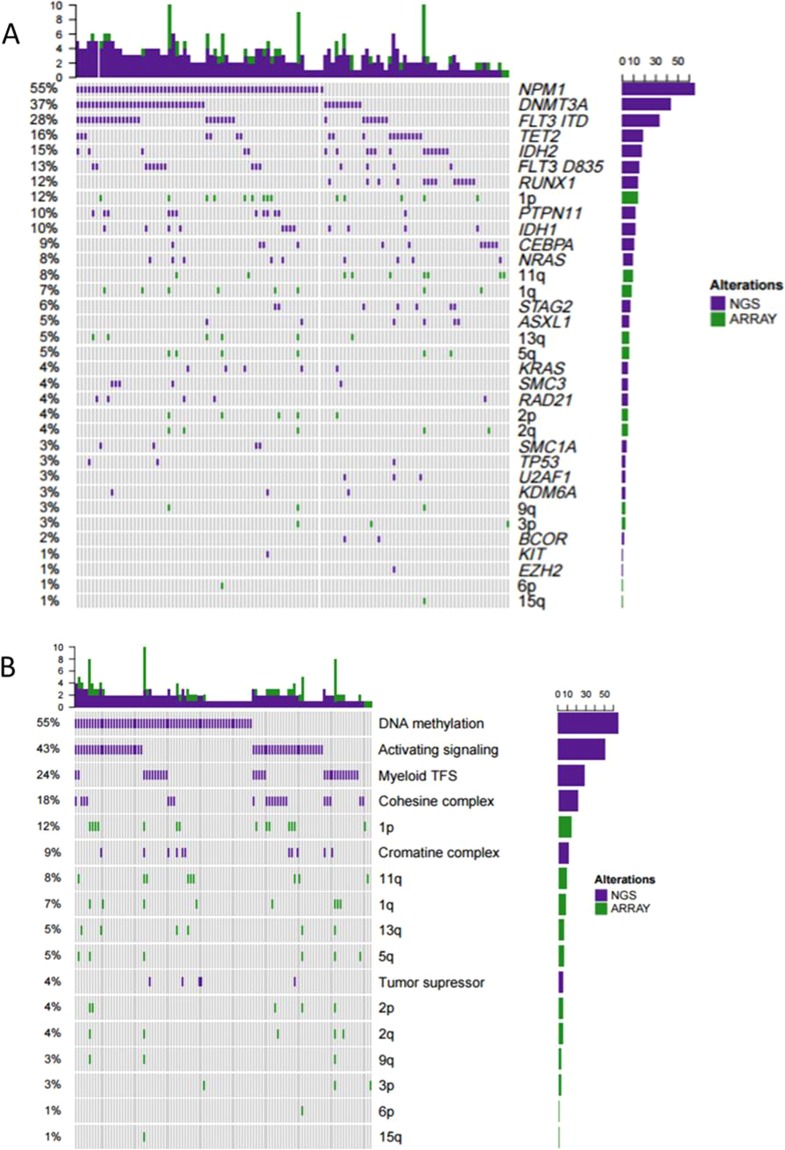
Distribution of cryptic cytogenetic alterations and mutations by order of frequency in patients with available data (N = 120). The bar graph at the top indicates the number of mutations per sample. The bar graph to the right shows the frequency of each mutation and reflects the numbers on its left side. (**A**) Cytogenetic alterations and genes mutated in order of frequency. (**B**) Cytogenetic alterations and genes categorized by oncogenic mechanism: DNA methylatiosn (*DNMT3A*, *IDH2, IDH1, TET2*); activating signaling (*KIT, KRAS, NRAS, PTPN11*); myeloid transcription factors (TFS) (*RUNX1, CEBPA, BCOR*); cohesin complex (*SMC1A, SMC3, STAG2, RAD21*); chromatic complex (*ASXL1, KDM6A, EZH2*); tumor suppressor (*TP53, U2AF1*).

In parallel, we analyzed NGS data from 120 patients with NK-AML and the results were correlated with SNP-A analyses displaying a pattern of association of those events. Distribution of cryptic cytogenetic alterations and mutations in order of frequency are shown in Fig. 3 (Supplementary Table 2). The most frequent mutated genes were *NPM1* (55%), *DNMT3A* (37%), *FLT3-ITD* (28%), *TET2* (16%), *IDH2* (15%), and *RUNX1* (12%). Then, we grouped the mutations by functional categories and observed that more than half of the cases had mutations in genes responsible for DNA methylation (55%) and/or in genes involved in cell signaling activation (43%). It should be noted that there was a significant number of patients with unique mutations in DNA methylation genes.

Regarding the patients harboring CNA or CN-LOH and point mutations simultaneously (n = 59), we found that 67% of patients with losses in chr 2p or 2q (n = 14) carried *DNMT3A* mutations, 75% with del(7q) or LOH7q (n = 16) were *EZH2*^pos^, 100% with del(13q) or LOH13q (n = 20) were *FLT3*^pos^, 67% with LOH19q (n = 16) were *CEBPA*^pos^ and 50% with LOH21q were *RUNX1*^pos^ (Supplementary Table 3). However, any statistically significant difference in the mutational profile was found between cases carrying and those lacking cryptic abnormalities.

### Correlation with clinical data

**Figure 4:**
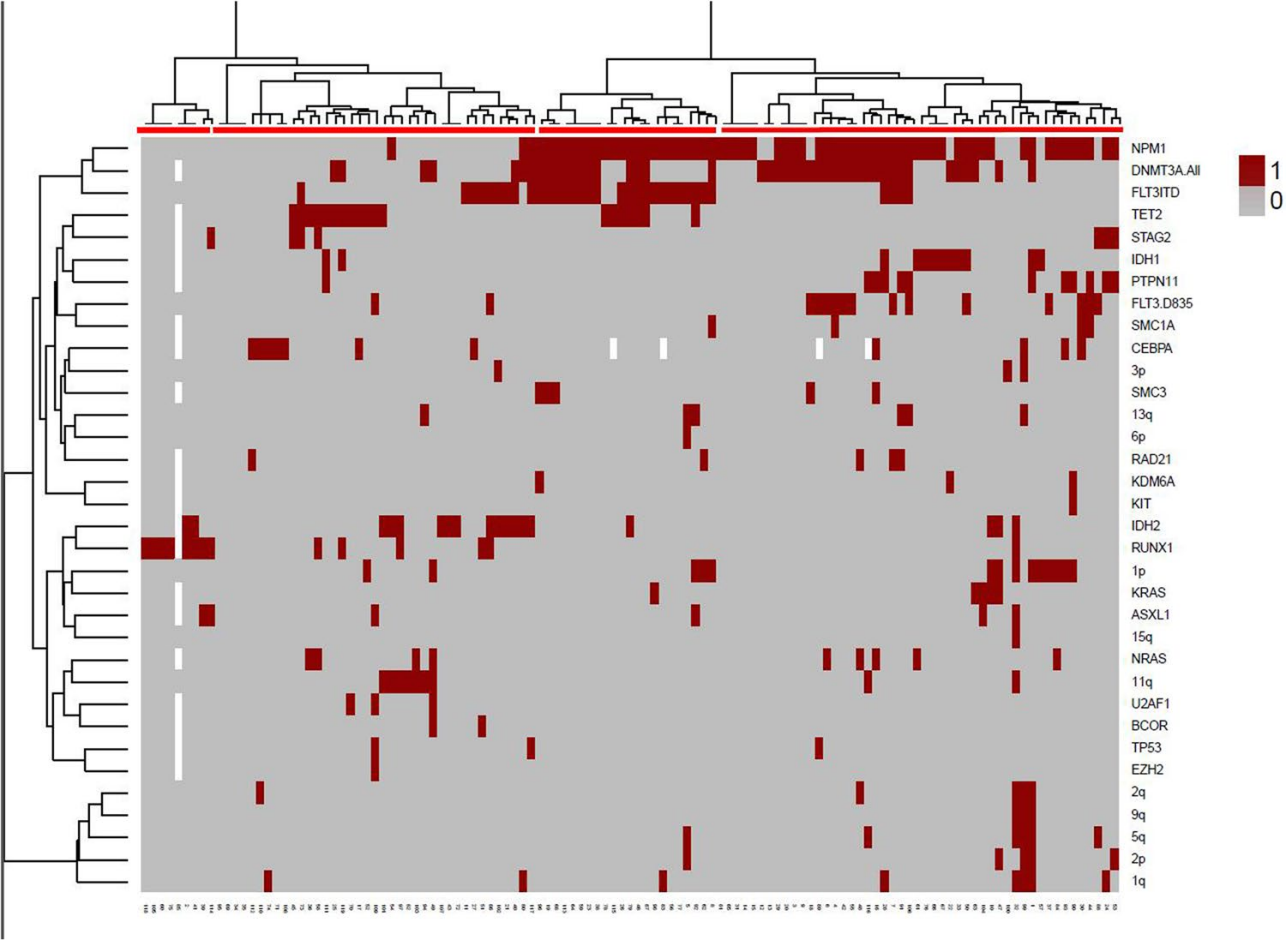
Heatmap depicting abnormalities found in our series. Each column represents one patient. Abnormalities are listed in the Y axis and coloured in the corresponding row of the heatmap. A red line delineates the clustered groups. 0 means negative; 1 means positive.

Patients were grouped as unsupervised clustering in 4 different sets according to their molecular abnormalities (Fig. 4): patients carrying several alterations simultaneously, characterized by *NPM1* and/or *DNMT3A* mutated, underlining its primary character in leukemogenesis (Cluster 1); patients harboring *RUNX1* mutations (Cluster 2); patients with mutations in *FLT3, NPM1* and/or *DNMT3A* (Cluster 3); and patients with a wide range of mutations, in which there was not a common pattern although *TET2* or *IDH2* were more frequently mutated, (Cluster 4). These sets showed different drivers of leukemogenesis in the 4 categories outlined.

**Figure 5:**
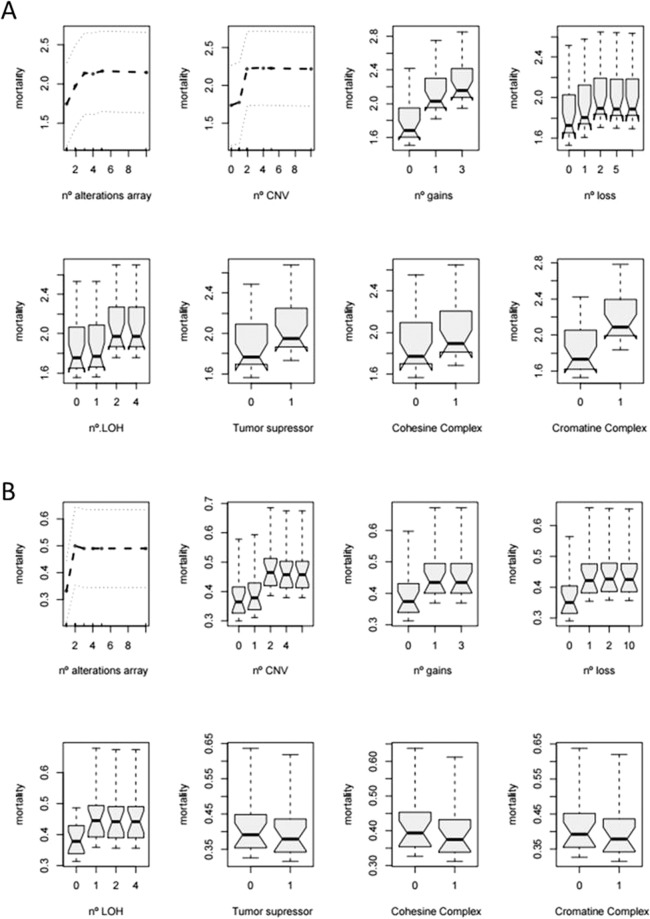
Partial dependence plots for each variable selected by Random Forest. The effect of each variable on survival while controlling for all the other variables is represented. (**A**) Overall Survival. (**B**) Relapse Free Survival.

The follow-up of the patients was updated on December 2017, and all follow-up data were censored at that time point. The median follow-up of surviving patients was 58 months (range, 26 to 124). Results from elastic net cox regression and random forest survival models did not find evidence of associations between any single CNA and clinical variables, even when we classified patients according to *FLT3*-ITD mutations. The elastic net analysis was not able to capture any association at all. Therefore, the null model was considered the optimum for this analysis. However, according to random forest results, the number of alterations detected in the array had an impact on the outcome of the patients; specifically, patients with 2 or more submicroscopic alterations had a worse overall survival (OS), being this effect more evident in gains and in CN-LOHs. It is worth highlighting that the presence of just one loss had a negative impact on the outcome, independently of the number of bases involved. The same was observed in terms of disease free survival and relapse free survival (DFS and RFS) (Fig. 5). Alterations in the cohesin and chromatin complexes also showed an association with lower OS. We also performed Cox regression models to assess the association between the 4 clusters of patients and survival. In this regard, our results show that Cluster 1 has a statistically significant higher OS compared to the other three clusters (Cluster 2 *vs*. Cluster 1: HR = 15.7, p = 0.025; Cluster 3 *vs*. Cluster 1: HR = 12.3, p = 0.022; Cluster 4 *vs*. Cluster 1: HR = 9.1, p = 0.039). Additionally, marginal estimated means on the log scale for death risk were estimated for each group (Cluster 1: −1.38, Cluster 2: 1.37, Cluster 3: 1.13, Cluster 4: 0.83). Regarding relapse free survival, no statistically significant differences were found among the different groups. (Cluster 2 *vs*. Cluster 1: HR = 2.4, p = 0.48; Cluster 3 *vs*. Cluster 1: HR = 3.0, p = 0.23; Cluster 4 *vs*. Cluster 1: HR = 1.7, p = 0.57). Marginal estimated means on the log scale for relapse risk were estimated for each group (Cluster 1: −0.52, Cluster 2: 0.34, Cluster 3: 0.58, Cluster 4: 0).

## Discussion

The present study shows that cryptic SNP abnormalities are present in the vast majority of *de novo* patients with NK-AML (58%), when analyzed by an ultra-high-density SNP-A karyotyping technique. In addition, their negative impact on the outcome of the patients is described. Finally, NGS data from 120 patients with NK-AML [LaFe cohort (n = 44) and TCGA cohort (n = 76)] was analyzed and correlated with SNP-A results, where 49% of patients harbored CNA or CN-LOH and point mutations simultaneously.

Many studies have demonstrated that lesions not detected by metaphase cytogenetics may be present in the samples and identified by an ultra-high-density SNP-A array in ~47% of *de novo* patients with NK-AML (Supplementary Table 4)^4–9,13,15^. This study comprises a cohort of 120 patients, of which more than half (n = 58%) showed cryptic SNP abnormalities. We extended our cohort to a total of 252 patients by comparing our data with that of 132 patients previously reported in other studies. To our knowledge, this is one of the few SNP array-based on genomics studies that has been performed using Cytoscan HD (Affymetrix), in which paired germline DNA was used^4,9–11,13,16,17^. Due to the absence of available paired samples from our expanded cohort, after a conversion of hg18 to version hg19, germline alterations were excluded from the analysis by visual inspection and by comparison with the polymorphic variations reported in the Database of Genomic Variants. Through this analysis, we were able to identify 282 cryptic somatically acquired losses, gains and CN-LOH in 146 patients. As expected, patients carried variable lesion loads, with an average of 2.32 abnormalities/patient (range 1–39), harboring some of them more than two cryptic aberrations at same time (n = 15). In our cohort, losses were more frequent than gains or CN-LOH (54%, 27% and 19% respectively) as well as in previous reports^4,9–11,13,16,17^. Losses were distributed virtually across all chromosomes, differently from CN-LOH or gains. The majority of lesions were not recurrent, with the exception of those located in 13q. Larger number of alterations were located in chr 1, 2, 11, 13, and 19 and distributed along genes that have previously showed to be involved in the pathogenesis of AML, such as *KMT2A, FLT3, ETV6, RUNX1* and *HNPRK*. Losses and CN-LOH were concentrated in chr 1, 2, 5q, 7, 11q and 13q. Due to the location and general implications of losses and CN-LOH, these abnormalities could be acting as a second-hit in the leukemogenesis process due to the loss of the wild-type allele.

SNP-A, NGS and clinical data were available for 120 patients, of which 49% harbored mutations and cryptic aberrations simultaneously. The most frequent mutated genes in those patients were *NPM1, DNMT3A, FLT3-ITD, TET2, IDH2* and *RUNX1*. Consistent with these studies, we identified mutations in *DNMT3A, EZH2, FLT3, CEBPA* and *RUNX1* in approximately two-thirds of cases analyzed by both CNAs [del(2p/2q); del(7q) or LOH7q; del(13q) or LOH13q; LOH19q or LOH21q, respectively] and targeted sequencing. These data suggest that alterations in a range of distinct biologic pathways might be cooperating with cryptic abnormalities to trigger leukemia. When mutations were grouped according to their functional category, we observed that nearly 50% of patients presented mutations in genes involved in DNA methylation and/or cell signaling activation. The analysis of the results led us to differentiate our cohort of patients in four distinct subsets: patients carrying more than one alteration simultaneously, characterized by *NPM1* and/or *DNMT3A* mutated, underlining its primary character in leukemogenesis (Cluster 1); patients harboring *RUNX1* mutations (Cluster 2); patients with mutations in *FLT3, NPM1* and/or *DNMT3A* co-occurring with many others (Cluster 3); and patients with a wide range of mutations without a recurrent pattern, although *TET2* or *IDH2* were more frequently mutated (Cluster 4). Any differences were detected in outcomes for the groups identified by unsupervised clustering. They were very similar, although there were a sizeable number of outliers in the first group. The proposed multivariate analysis was very limited because the variables were highly imbalanced (*RUNX1*: 14 positives *vs*. 104 negatives; *TP53*: 3 positives *vs*. 115 negatives; *ASXL1*: 8 positives *vs*. 110 negatives). Nevertheless, we performed the analysis and found that there is a significant association between cluster 2 and *RUNX1* (All patients in cluster 2 are positive for *RUNX1*, p < 0.001). We also performed cox regression models to assess the association between the 4 clusters of patients and survival. In this regard, our results show that cluster 1 has a statistically significant higher overall survival compared to the other three clusters. However, no statistically significant differences were found regarding relapse free survival. In previous reports, the presence of abnormal SNP-A detected lesions has an adverse impact on clinical outcome and is associated with disease progression^4,9–11,13,16,17^. Consistent with these results, in this study the number of cryptic abnormalities seemed to have an adverse impact on the final outcome. In fact, the presence of ≥2 genomic lesions had a negative impact on patient survival, although this must be cautiously interpreted due to the relatively small cohort analyzed. Larger AML cohorts would be needed to elucidate the impact on genomic complexity and the individual recurrent genomic abnormalities on the clinical outcome classification.

The main limitation of our study is the selection bias of our patients. We have mainly analyzed patients from two different sources, the Spanish selection and the TCGA [LaFe cohort (n = 44) and TCGA cohort (n = 76)]. The Spanish patients were enrolled in consecutive multicenter PETHEMA trials. However, there was not sufficient information available on the treatment administered to TCGA patients. Statistical analysis confirmed that there were no differences between both series of patients, reflecting the suitability of pooling both cohorts in a unique study. Nevertheless, it must be noticed that the analyzed patients were not randomly included in our study, owing the difficulties encountered when setting up a significant independent association with the outcome, using univariate or multivariate analysis. The same limitations were met when analyzing the expanded cohort. In addition, different density SNP-A has been used among the studies. That could cause a loss of some recurrent cryptic abnormalities trailing their effect on the outcome of the patients. Finally, other limitation was the breakdown for copy number changes that was considered truly cryptic. We include all those aberrations not previously detected by daily cytogenetic test, such as G-Banding pattern and FISH, (placed conservatively at 20 Mb). Thus, included aberrations could be changes that were not truly cryptic but rather reflect failure to detect the abnormal clone by cytogenetic technique because of reduced representation of a clone with microscopically detectable changes in the dividing cell fraction.

## Conclusion

In summary, our data demonstrated that more than half of the patients with NK-AML harbored cryptic SNP abnormalities which had a negative impact on their outcome. As a result, the use of ultra-high-resolution SNP arrays can be considered as an additional tool for better prognostic stratification of patients with NK-AML, to enable the detection of cryptic aberrations that add information to AML diagnosis. However, more data are necessary to support the implementation of SNP-A in the routine diagnosis, especially in the context of targeted therapies.

## Supplementary information

Supplementary Information.

## Data Availability

Main data generated or analysed during this study are included in this published article [and its supplementary information files]. The arrays files and clinical data analysed during the current study are available from the corresponding author on reasonable request.
