# Supplementary material for: Analysis of SNP Array Abnormalities in Patients with DE NOVO Acute Myeloid Leukemia with Normal Karyotype
Source: Sci Rep. 2020 Apr 3;10:5904. doi: 10.1038/s41598-020-61589-9 (PMC7125150; doi:10.1038/s41598-020-61589-9)
Supplement: Supplementary file 1 — Supplementary Information. [file 41598_2020_61589_MOESM1_ESM.pdf]

ANALYSIS OF SNP ARRAY ABNORMALITIES IN PATIENTS WITH *DE NOVO* ACUTE MYELOID LEUKEMIA WITH NORMAL KARYOTYPE

Mariam Ibáñez, [1,2,3] Esperanza Such, [1,2] Esther Onecha,[4] Inés Gómez-Seguí, [1,2] Alessandro Liquori, [2,5] Jorge Sellés,[6] David Hervás-Marín,[7] Eva Barragán, [2,8] Rosa Ayala,[3] Marta LLop,[2,8] María López-Pavía,[9] Inmaculada Rapado,[3] Alex Neef,[3] Alejandra Sanjuan-Pla,[3] Claudia Sargas,[3,8] Elisa Gonzalez-Romero,[3] Mireia Boluda-Navarro,[3] Rafa Andreu, [1] Leonor Senent, [1,2] Pau Montesinos, [1,2] Joaquín Martínez-López,[3] Miguel Angel Sanz, [1,2,10] Guillermo Sanz [1,2] and José Cervera [1,2,11]

[1]Hematology Service, Hospital Universitario y Politécnico La Fe, Valencia, Spain; [2]Centro de Investigación Biomédica en Red de Cáncer; [3] Departamento de Ciencias Biomédicas. Facultad de Ciencias de la Salud. Universidad CEU Cardenal Herrera. Valencia, Spain; [4]Hospital 12 de Octubre, Madrid, Spain; [5]Grupo de Investigación en Hematología, IIS La Fe, Valencia, Spain; [6]Array's Unit. Instituto Investigación Sanitaria Fundación La Fe, Valencia, Spain; [7]Biostadistic Unit. Instituto Investigación Sanitaria Fundación La Fe, Valencia, Spain; [8]Laboratory of Molecular Biology, Department of Clinical Chemistry, University Hospital La Fe, Valencia, Spain; [9]Hematology Service, Hospital General, Valencia, Spain; [10]Department of Medicine. University of Valencia, Valencia, Spain; [11]Genetics Unit, Hospital Universitario y Politécnico La Fe, Valencia, Spain.

Corresponding author:

José Cervera
Hospital Universitari i Politécnic La Fe
Avinguda de Fernando Abril Martorell, nº 106
46026 Valencia
0034961 244688
E-mail: cervera_jos@gva.es

Running title: SNP-A Karyotyping in AML with normal karyotype

Key words: SNP-A, AML, normal karyotype, NGS

Word count: Manuscript: 2732 words; Abstract: 245 words.

SUPPLEMENTARY

Supplementary Table 1. Comparison of SNP-A abnormalities in reported AML series

Supplementary Table 2. Cryptic cytogenetic alterations and mutations detected in our series (n=120)

Supplementary Table 3. Concurrence of CNA or CN-LOH and point mutations in NK-AML

Supplementary Table 4. Comparison of previous reports in AML

Supplementary Table 1. Comparison of SNP-A abnormalities in reported AML series

Study	No. of cases with NK	Paired sample	Microarray Platform	Patients harbouring alterations	No. detected alterations (selected)
Ibáñez M, et al.	44	Yes	Cytoscan HD (Affymetrix)	57%	55 (55)
Krönke J, et al.	53	Yes	50k/500k SNP (Affymetrix)	25%	15 (8)
Agaki T, et al.	30	No	250 K SNP (Affymetrix)	60%	25 (15)
Koren-Michowitz M, et al.	49	No	250 K SNP (Affymetrix)	84%	74 (61)
TCGA	76	Yes	6.0 SNP (Affymetrix)	56%	210 (143)
**Total**	**252**			146	

Supplementary Table 2 . Cryptic cytogenetic alterations and mutations deteted in our series (n=120)

id	Cohort	No. Alterations	Gains	Losses	LOH	*KIT*	*KRAS*	*NRAS*	*PTPN11*	ACTIVATINGSI	*FLT3D835*	*FLT3ITD*	*NPM1*	*DNMT3A*	*IDH2*	*IDH1*	*TET2*	DNA METHYL	*RUNX1*	*CEBPA*	*BCOR*	MYELOID TFS	*TP53*	*U2AF1*	tumor Supres	*SMC1A*	*SMC3*	*STAG2*	*RAD21*	Cohesine Com	*ASXL1*	*KDM6A*	*EZH2*	CROMATINE C
#12	M Ibañez et al.	2	0	2	0	0	0	0	0	0	0	1	0	0	1	0	0	1	0	0	0	0	0	0	0	0	0	0	0	0	0	0	0	0
#14	M Ibañez et al.	2	1	1	0	0	0	1	0	1	0	0	0	0	0	0	0	0	0	0	0	0	0	0	0	0	0	0	0	0	0	0	0	0
#17	M Ibañez et al.	1	1	0	0	0	0	0	0	0	0	0	0	0	1	0	1	1	0	0	0	0	0	0	0	0	0	0	0	0	0	0	0	0
#24	M Ibañez et al.	2	0	1	1	0	0	0	0	0	0	0	0	0	0	0	0	0	0	0	0	0	0	0	0	0	0	0	0	0	0	0	0	0
#26	M Ibañez et al.	1	0	0	1	0	0	0	1	1	0	0	1	0	0	0	0	0	0	1	0	1	0	0	0	0	0	0	0	0	0	0	0	0
#27	M Ibañez et al.	1	0	1	0	0	0	0	1	1	0	0	1	0	0	0	0	0	0	0	0	0	0	0	0	0	0	0	0	0	0	0	0	0
#28	M Ibañez et al.	1	0	1	0	0	0	0	0	0	0	0	0	0	1	0	0	1	0	0	0	0	0	0	0	0	0	0	0	0	0	0	0	0
#31	M Ibañez et al.	5	0	5	0	0	0	0	0	0	0	0	0	1	0	0	0	1	0	0	0	0	0	0	0	0	0	0	0	0	0	0	0	0
#32	M Ibañez et al.	1	0	0	1	0	0	0	1	1	1	1	1	1	0	0	0	1	0	0	0	0	0	0	0	0	0	0	0	0	2	0	0	1
#34	M Ibañez et al.	1	0	1	0	0	0	0	0	0	1	0	0	0	0	0	0	0	0	0	0	0	0	0	0	0	0	1	0	1	0	0	0	0
#35	M Ibañez et al.	1	1	0	0	0	0	0	0	0	0	0	0	0	0	0	0	0	0	0	0	0	0	0	0	0	0	0	0	0	0	0	0	0
#36	M Ibañez et al.	1	0	0	1	0	0	0	1	1	0	1	1	1	0	0	0	1	0	0	0	0	0	0	0	0	0	0	1	1	0	0	0	0
#39	M Ibañez et al.	1	0	1	0	0	0	0	0	0	1	0	1	1	0	0	0	1	0	NA	0	1	1	0	1	0	0	0	0	0	0	0	0	0
#42	M Ibañez et al.	2	0	0	2	1	0	0	1	1	0	0	1	0	0	0	0	0	0	0	0	0	0	0	0	0	0	0	0	0	0	1	0	1
#44	M Ibañez et al.	2	0	2	0	0	0	0	0	0	0	0	1	1	1	0	0	1	0	0	0	0	0	0	0	0	0	0	0	0	0	0	0	0
#46	M Ibañez et al.	10	0	10	0	0	0	0	0	0	0	0	1	0	0	0	0	0	0	1	0	1	0	0	0	0	0	0	0	0	0	0	0	0
#47	M Ibañez et al.	5	3	1	1	0	0	0	0	0	0	0	0	0	0	0	0	0	0	1	0	1	0	0	0	0	0	0	0	0	0	0	0	0
#49	M Ibañez et al.	2	1	0	1	0	0	0	0	0	0	0	0	0	1	0	0	1	1	0	0	1	0	0	0	0	0	0	0	0	0	0	0	0
#50	M Ibañez et al.	3	0	1	2	0	0	0	0	0	0	0	0	0	0	0	0	0	1	0	0	1	0	0	0	0	0	1	0	1	1	0	0	1
#54	M Ibañez et al.	1	0	0	1	0	0	0	0	0	0	1	1	0	0	0	0	0	0	NA	0	1	0	0	0	0	0	0	0	0	0	0	0	0
#55	M Ibañez et al.	1	1	0	0	0	0	0	0	0	1	0	0	0	0	0	1	1	0	0	0	0	1	1	1	0	0	0	0	0	1	0	1	1
#56	M Ibañez et al.	1	0	0	1	0	0	1	0	1	0	0	1	0	0	0	0	0	0	0	0	0	0	0	0	0	0	0	0	0	2	0	0	1
#57	M Ibañez et al.	4	0	0	4	0	0	0	1	1	0	0	1	1	0	0	0	1	0	NA	0	1	0	0	0	0	0	0	0	0	0	0	0	0
#8	M Ibañez et al.	4	0	2	2	0	0	0	0	0	0	1	1	0	0	0	1	1	0	0	0	0	0	0	0	0	0	0	0	0	1	0	0	1
2802	TCGA	39	6	33	0	0	0	0	1	1	0	0	1	1	0	1	0	1	0	0	0	0	0	0	0	0	0	0	0	0	0	0	0	0
2811	TCGA	5	4	1	0	0	0	0	0	0	1	0	1	1	0	0	0	1	0	0	0	0	0	0	0	1	0	0	0	1	0	0	0	0
2812	TCGA	6	3	3	0	0	0	0	0	1	0	1	1	0	0	0	0	0	0	0	0	0	0	0	0	0	0	0	0	0	0	0	0	0
2824	TCGA	1	0	1	0	0	0	0	0	1	0	1	1	1	0	0	0	0	0	0	0	0	0	0	0	1	0	0	0	1	0	0	0	0
2825	TCGA	1	0	1	0	0	0	0	0	1	0	0	1	1	0	0	0	1	0	0	0	0	0	0	0	0	0	0	0	0	0	0	0	0
2826	TCGA	1	1	0	0	0	1	0	0	1	0	0	1	0	1	0	0	1	0	0	0	0	0	0	0	0	0	0	0	0	0	0	0	0
2831	TCGA	1	0	1	0	0	0	0	0	0	0	0	0	1	0	0	0	0	0	0	0	0	0	0	0	0	0	0	0	0	0	0	0	0
2833	TCGA	1	1	0	0	0	0	0	0	1	0	0	0	1	0	0	0	1	0	0	0	0	0	0	0	0	0	0	0	0	0	0	0	0
2866	TCGA	1	1	0	0	0	0	0	0	0	0	1	0	0	1	0	0	1	0	0	0	0	0	0	0	0	0	0	0	0	0	0	0	0
2871	TCGA	5	4	0	1	0	0	0	1	1	0	0	1	0	0	0	0	0	0	0	0	0	0	0	0	0	0	1	0	1	0	0	0	0
2879	TCGA	1	1	0	0	0	0	0	0	1	0	1	1	0	0	0	1	1	0	0	0	0	0	0	0	0	0	0	0	0	0	0	0	0
2884	TCGA	1	0	1	0	0	0	0	1	1	0	1	1	1	0	1	0	1	0	0	0	0	0	0	0	0	0	0	0	0	0	0	0	0
2896	TCGA	1	0	1	0	0	0	0	0	0	0	0	1	1	0	0	0	1	0	0	0	0	0	0	0	0	0	0	0	0	0	0	0	0
2907	TCGA	23	10	13	0	0	0	0	0	0	0	0	0	0	1	0	0	1	1	0	0	1	0	0	0	0	0	0	0	0	1	0	0	1
2919	TCGA	5	5	0	0	0	0	0	0	1	0	0	1	1	0	1	0	1	0	0	0	0	0	0	0	0	0	0	0	0	0	0	0	0
2921	TCGA	2	0	2	0	0	0	0	0	1	0	0	0	0	0	0	0	0	0	0	0	0	0	0	0	0	0	0	0	0	0	0	0	0
2922	TCGA	1	0	1	0	0	0	0	0	1	0	0	0	0	0	0	0	0	0	0	0	0	0	0	0	0	0	0	0	0	0	0	0	0
2924	TCGA	1	0	0	1	0	0	0	0	0	1	0	1	0	0	0	0	0	0	0	0	0	0	0	0	0	0	0	0	0	0	0	0	0
2934	TCGA	1	0	1	0	0	0	0	0	1	0	1	0	1	1	0	0	1	0	0	0	0	0	0	0	0	0	0	0	0	0	0	0	0
2964	TCGA	2	1	0	1	0	0	0	0	0	0	0	0	0	0	0	1	1	0	0	0	0	0	0	0	0	0	1	0	1	0	0	0	0
2966	TCGA	3	2	1	0	0	1	0	0	1	0	0	0	1	1	0	0	1	0	0	0	0	0	0	0	0	0	0	0	0	0	0	0	0
2967	TCGA	1	0	1	0	0	0	1	0	1	0	0	1	1	0	0	0	0	0	0	0	0	0	0	0	0	0	0	1	1	0	0	0	0
2968	TCGA	2	1	1	0	0	0	1	0	1	0	0	0	1	0	0	0	0	0	0	1	0	0	1	1	0	0	0	0	0	0	0	0	0
2970	TCGA	1	0	0	1	0	0	0	0	1	0	1	0	0	0	0	0	0	1	0	1	1	0	0	0	0	0	0	0	0	0	0	0	0
2971	TCGA	1	1	0	0	0	0	0	0	0	0	0	0	0	0	0	1	1	0	0	0	0	0	0	0	0	0	0	0	0	0	0	0	0
2972	TCGA	1	1	0	0	0	0	0	1	1	0	0	1	0	0	0	0	0	0	0	0	0	0	0	0	0	0	1	0	1	0	0	0	0
2973	TCGA	3	0	3	0	0	0	0	0	0	0	0	1	0	1	0	0	1	0	0	0	0	0	0	0	0	0	0	0	0	0	0	0	0
2974	TCGA	1	0	1	0	0	0	0	0	0	1	0	1	1	0	0	0	1	0	0	0	0	0	0	0	0	0	0	0	0	0	0	0	0
2976	TCGA	1	0	1	0	0	0	0	0	1	0	1	1	0	0	0	0	0	0	0	0	0	0	0	0	0	0	0	0	0	0	0	0	0
2977	TCGA	2	1	1	0	0	0	0	0	1	0	0	0	0	0	1	0	1	0	0	0	0	0	0	0	0	0	0	0	0	0	0	0	0
2983	TCGA	2	0	1	1	0	0	0	0	0	0	0	0	0	0	0	0	0	1	0	0	1	0	0	0	0	0	0	0	0	0	0	0	0
2986	TCGA	1	1	0	0	0	0	0	0	1	0	1	1	0	0	0	0	0	0	0	0	0	0	0	0	0	0	0	1	1	0	0	0	0
2987	TCGA	2	0	2	0	0	1	0	0	1	0	0	1	1	0	0	0	1	0	0	0	0	0	0	0	0	0	0	0	0	0	0	0	0
2989	TCGA	1	0	1	0	0	0	0	0	0	0	0	1	0	0	0	0	0	0	0	0	0	0	0	0	0	0	0	0	0	0	0	0	0
2992	TCGA	1	0	1	0	0	0	0	0	0	0	0	1	0	0	1	0	1	0	0	0	0	0	0	0	0	0	0	0	0	0	0	0	0
3006	TCGA	1	1	0	0	0	0	0	0	1	0	1	0	0	0	0	1	1	0	0	0	0	0	0	0	0	0	1	0	1	0	0	0	0
3008	TCGA	2	0	2	0	0	0	0	0	0	0	0	0	0	0	0	0	0	0	1	0	1	0	0	0	0	0	0	0	0	0	0	0	0
3009	TCGA	2	2	0	0	0	0	0	0	0	0	0	0	0	0	0	0	0	1	0	0	1	0	0	0	0	0	0	0	0	0	0	0	0

Supplementary Table 3. Concurrence of CNA or CN-LOH a

Region	Nº alterated patientes	Mutated gene	% patients harbouring mutations in
del(2p)<br>Del(2q)	14	*DNMT3A*	67%
del(7q)<br>LOH7q	16	*EZH2*	75%
LOH11p	3	*WT1, ATM*	NA
del(11q)<br>LOH11q	9	*KMT2A*	NA
del(13q)<br>LOH13q	20	*FLT3*	100%
LOH19q	3	*CEBPA*	67%
LOH21q	2	*RUNX1*	50%

*Data from TCGA and Ibáñez et al.

Supplementary Table 4. Comparison of previous reports in AML

Study	AML Subtypes	No. of cases with NK	Microarray Platform	Normal paired samples	Conclusions
Tyybakinoja et al.	NK-AML	26	44k CGH (Agilent)	No	Detection of submicroscopic genomic deletions and gains
Walter et al.	All citogenetic groups	34	6.0 SNP (Affymetrix)	Paired samples (matched skin biospies)	24% of NK patients had CNA, losses more recurrent; 15% pa showed LOH
Agaki et al.	NK-AML	30	250 K SNP (Affymetrix)	No	CNA in 60% patients. Losses in *NF1, ETV6, CDKN2A*. Gains mainly at chr 8. 32% patients showed LOH
Bullinguer et al.	NK-AML	159	50k/500k SNP (Affymetrix)	Paired samples (matched remission)	CNAs in 49% of cases, detection of cryptic translocations, correlation of UPD with outcome
Parkin et al.	All citogenetic groups	25	6.0 SNP (Affymetrix)	Paired germline DNA (buccal swab DNA)	Poor outcome in cases with ≥2 genomic lesions, negative prognostic impact of UPD17p
Yi et al.	NK-AML	133	6.0 SNP (Affymetrix)	No	SNP lesions detected by SNP-Array indicates a worse outcome, with the exception of patients with *FLT3* -ITD mutated
Koren-Michowitz et al.	NK-AML	49	250 K SNP (Affymetrix)	No	Older patients showed more abnormalities. SNP lessions worse OS
Krönke et al.	All citogenetic groups	44	50k/500k SNP (Affymetrix)	Paired relapse sample	At diagnosis time, no recurrent aberrations with the exception of del(9) and UDP13q
TCGA et al.	All citogenetic groups	79	6.0 SNP (Affymetrix)	Paired normal skin samples	Low frequency of criptic alteraions in NK-AML patientes. Losses more frequent than gains
Nibourel et al.	All citogenetic groups	178	6.0 SNP (Affymetrix)	Only in 50 patients paired samples (matched remission)	Chromosomes 11 and 21sowed a poor outcome with independent prognostic relevance
